# Comparison of Cytotoxic, Genotoxic, and DNA-Protective Effects of Skyrin on Cancerous vs. Non-Cancerous Human Cells

**DOI:** 10.3390/ijms23105339

**Published:** 2022-05-10

**Authors:** Terézia Zajičková, Eva Horváthová, Stanislav Kyzek, Eva Šályová, Eva Túryová, Andrea Ševčovičová, Eliška Gálová

**Affiliations:** 1 Department of Genetics, Faculty of Natural Sciences, Comenius University in Bratislava, Ilkovičova 6, 842 15 Bratislava, Slovakia; zajickova21@uniba.sk (T.Z.); eva.salyova@img.cas.cz (E.Š.); turyova10@uniba.sk (E.T.); andrea.sevcovicova@uniba.sk (A.Š.); eliska.galova@uniba.sk (E.G.); 2 Cancer Research Institute, Biomedical Research Centre of the Slovak Academy of Sciences, Dúbravská Cesta 9, 845 05 Bratislava, Slovakia; eva.horvathova@savba.sk

**Keywords:** skyrin, genotoxicity, cytotoxicity, DNA-protectivity, comet assay, HepG2 cells, lymphocytes

## Abstract

Secondary metabolites as a potential source of anticancer therapeutics have been the subject of many studies. Since hypericin, a metabolite isolated from *Hypericum perforatum* L., shows several biomedical properties applicable in oncology, the aim of our study was to investigate its potential precursor skyrin in terms of genotoxic and DNA-protective effects. These skyrin effects were analyzed by cell-free methods, and cytotoxicity was estimated by an MTT assay and by a trypan blue exclusion test, while the genotoxic/antigenotoxic potential was examined by comet assay using non-cancerous human lymphocytes and the HepG2 cancer cell line. Skyrin did not show DNA-damaging effects but rather exhibited DNA-protectivity using a DNA-topology assay. However, we observed only weak antioxidant and chelating skyrin properties in other cell-free methods. Regarding the cytotoxic activity of skyrin, HepG2 cells were more prone to skyrin-induced death in comparison to human lymphocytes. Skyrin in non-cytotoxic concentrations did not exhibit elevated genotoxicity in both cell types. On the other hand, skyrin displayed moderate DNA-protective effects that were more noticeable in the case of non-cancerous human lymphocytes. The potential genotoxic effects of skyrin were not observed, and its DNA-protective capacity was more prominent in non-cancerous cells. Therefore, skyrin might be a promising agent used in anticancer therapy.

## 1. Introduction

The plant kingdom is a great source of medicinal herbs that, due to their positive health effects, engaged the attention of people many years ago. Currently, thanks to previous empirical experience and traditional medicine, we have access to widespread biomedical information about numerous plants. Undoubtedly, St. John’s wort (*Hypericum perforatum* L.) is one of such examples. This medical plant has earned the attention of the scientific as well as the public community as a result of its wide-ranging use due mainly to the synergistic interactions of its secondary metabolites [1]. *Hypericum* extracts can be used as a cure for various diseases, including the treatment of wounds [2], burns [3], gastrointestinal problems [4], depression [5], and many others [6]. The medicinal herb *H. perforatum* L. contains a complex of various secondary metabolites characterized by specific properties with potential application in the pharmaceutical industry and medicine. Therefore, extensive research focused on the genotoxic profiling of its individual compounds, such as hypericin [7,8], emodin [9], hyperforin, and aristoforin [10,11], was performed.

Hypericin, the most well-known metabolite of *H. perforatum* L., is one of the most promising agents for anticancer photodynamic therapy [12,13,14,15] due to its photoactivation ability. Extensive commercial and scientific efforts to fully characterize this metabolite led to an investigation of its biosynthesis and the subsequent identification of skyrin, the potential precursor of hypericin in St. John’s wort [16,17]. Skyrin, a red pigment from the group of anthraquinones, is a secondary metabolite isolated not only from plants but also from insects and fungi [18,19]. Skyrin has been the subject of several studies mainly due to its association with hypericin. The ability of a selective accumulation of skyrin in necrotic tissue could possibly play a role in the diagnostics and treatment of tumors [20,21]. The proposal of skyrin as a prospective drug for the treatment of diabetes mellitus has been made since it acts as a receptor-selective glucagon antagonist, ultimately inhibiting gluconeogenesis and glycogenolysis and, thus, maintaining normal blood sugar levels [22]. The antibacterial properties of this chemical also increase its potential for clinical use [23,24,25,26]. Due to the fact that several medically beneficial compounds display genotoxic potentials [9], the effect of skyrin on DNA should be investigated before its future implementation into clinical practice. Since the effect of skyrin on various model systems has not been sufficiently explored, the aim of this study is to monitor its potential toxicity and genotoxicity using different models. Moreover, the potential mechanism of skyrin action was studied by cell-free methods.

## 2. Results

### 2.1. DNA-Damaging and DNA-Protective Effects of Skyrin on Plasmid DNA

**Figure 1:**
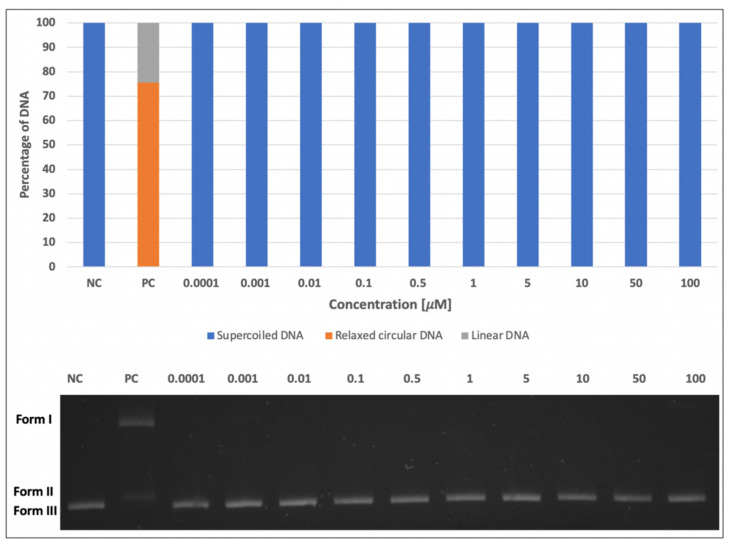
Potential DNA-damaging effects of skyrin on plasmid DNA. Electrophoretic monitoring of changes in the topology of plasmid DNA (pBR322) induced by skyrin (SK) using DNA-topology assay (Form I = relaxed circular DNA; Form II = linear DNA; Form III = supercoiled DNA). Legend: Line 1: pBR322 (negative control-NC); line 2: pBR322 + FeSO_4_ × 7H_2_O (positive control-PC); lines 3–12: pBR322 + different concentrations of skyrin. Experiments were performed three times with similar results, and the representative electrophoretic gel is presented. The bar chart shows the percentage of individual topological forms of plasmid DNA in corresponding lines.

**Figure 2:**
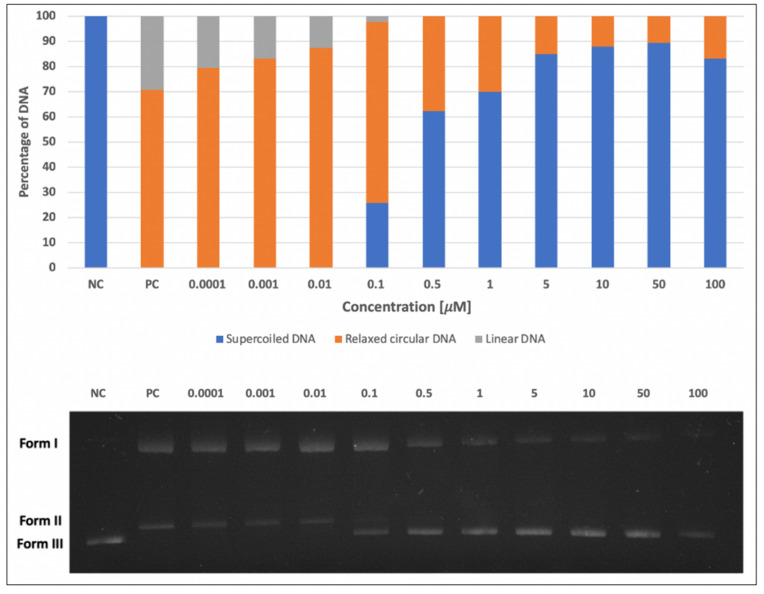
DNA-protective activity of skyrin (SK) analyzed by DNA-topology assay. Electrophoretic monitoring of changes induced by FeSO_4_ × 7H_2_O in the topology of plasmid DNA (pBR322) after skyrin application (Form I = relaxed circular DNA; Form II = linear DNA; Form III = supercoiled DNA). Legend: Line 1: pBR322 (negative control-NC); line 2: pBR322 + FeSO_4_ × 7H_2_O (positive control-PC); lines 3–12: pBR322 + FeSO_4_ × 7H_2_O + different concentrations of skyrin. Experiments were performed three times with similar results, and the representative electrophoretic gel is presented. The bar chart shows the percentage of individual topological forms of plasmid DNA in corresponding lines.

The effects of skyrin on plasmid DNA were investigated by DNA-topology assay. Firstly, we determined the potential of skyrin to form single- and double-strand breaks in plasmid DNA. None of the tested concentrations of skyrin (0.0001–100 μM) exhibited DNA-damaging properties, similarly to the negative control (Figure 1). The potential DNA-protective capacity against Fe^2+^-induced DNA breaks was also evaluated. The ability of skyrin to protect plasmid DNA increased in a concentration-dependent manner (Figure 2), and strong DNA protectivity was observed in samples with higher concentrations of skyrin (5–100 μM).

### 2.2. Antioxidant and Chelating Potential of Skyrin in Cell-Free Methods

**Figure 3:**
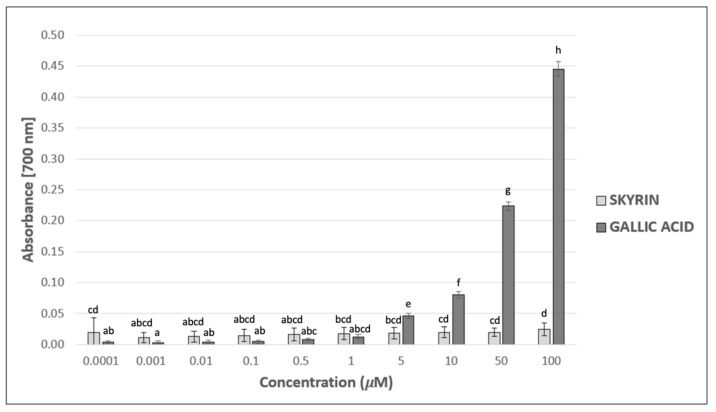
Reducing power of skyrin (light grey columns) in comparison with gallic acid used as a positive control at the same concentrations (dark grey columns). Each value is expressed as mean ± standard deviation. Results were analyzed by ANOVA single-step multiple comparisons of means using LSD tests, and comparisons between the mean values were considered significant at *p* < 0.05. Similar letters represent samples with no statistically significant differences between them. All the experimental data in this work are from at least three independent experiments.

**Figure 4:**
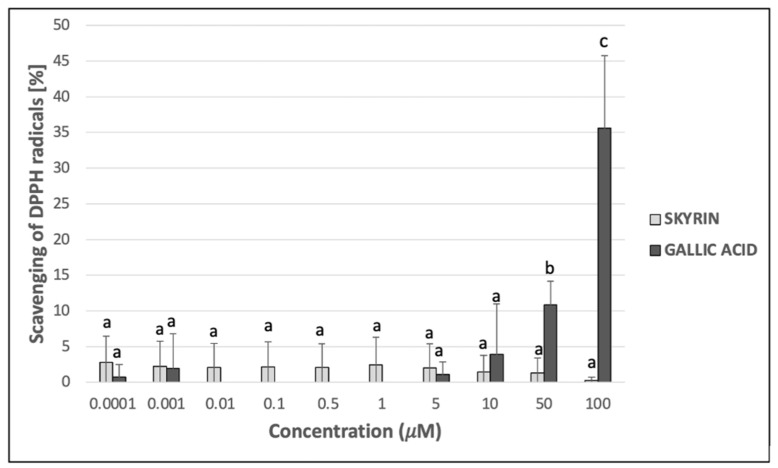
DPPH^•^ radical scavenging activity of skyrin (light grey columns) in comparison with gallic acid used as a positive control at the same concentrations (dark grey columns). Each value is expressed as mean ± standard deviation. Results were analyzed by ANOVA single-step multiple comparisons of means using LSD tests, and comparisons between the mean values were considered significant at *p* < 0.05. Similar letters represent samples with no statistically significant differences between them. All experimental data in this work are from at least three independent experiments.

**Figure 5:**
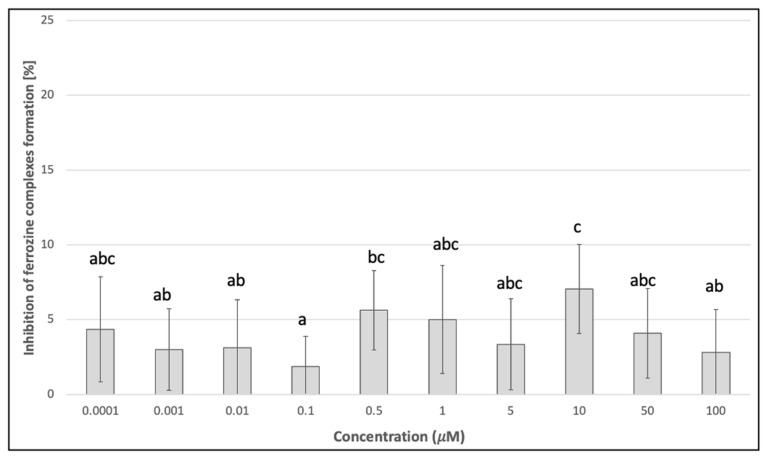
Chelating activity of skyrin. Each value is expressed as mean ± standard deviation. Results were analyzed by ANOVA single-step multiple comparisons of means using LSD tests, and comparisons between the mean values were considered significant at *p* < 0.05. Similar letters represent samples with no statistically significant differences between them. All experimental data in this work are from at least three independent experiments.

Since skyrin displayed protective effects against Fe^2+^-induced DNA breaks in plasmid DNA, its potential mechanism of action was investigated using other cell-free methods. The ability to donate electrons was estimated, using reducing power assay as an indicator of the potential antioxidant capacity of skyrin. However, skyrin did not show considerable reducing power compared to gallic acid (positive control), which was mainly noticeable at higher concentrations (Figure 3). Another possible antioxidant effect mechanism is the ability of the chemical to act as a hydrogen atom donor, thus interacting with radicals and decreasing their systemic levels. Skyrin in a wide range of concentrations (0.0001–100 μM) had only a slight ability to scavenge DPPH^•^ radicals, and the percentage of scavenged DPPH^•^ radicals in skyrin samples did not surpass the 5% level (Figure 4). Similar results were obtained in Fe^2+^-chelating assays, where the level of inhibition of ferrozine complexes formation did not exceed 10% in samples with skyrin (Figure 5). Based on these results, we concluded that skyrin exhibited very weak antioxidant or chelating capacities as detectable by the methods mentioned above.

### 2.3. Cytotoxic and Genotoxic Effects of Skyrin in Human Cells

**Figure 6:**
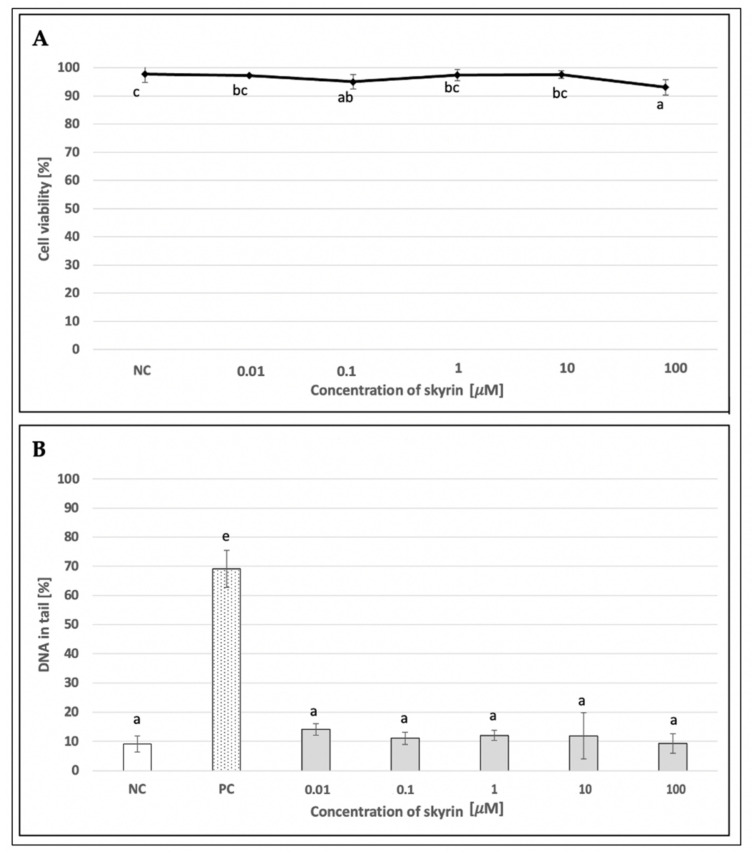
Evaluation of the potential cytotoxic (**A**) and genotoxic (**B**) effects of skyrin (SK) on human lymphocytes (1 h treatment) at different concentrations, using the trypan blue exclusion test and the comet assay. Negative control (NC)—untreated cells; positive control for comet assay (PC)—cells treated with 440 μM hydrogen peroxide. Results were analyzed by ANOVA single-step multiple comparisons of means using LSD tests, and comparisons between the mean values were considered significant at *p* < 0.05. Similar letters represent samples with no statistically significant differences between them. All experimental data in this work are from at least three independent experiments.

To evaluate the potential cytotoxic properties of skyrin on human lymphocytes, a trypan blue exclusion test was performed. Since cell viability in all skyrin samples was over 90% (Figure 6A), these results suggest that skyrin did not exert cytotoxic effects on human lymphocytes at the analyzed concentrations (0.01–100 μM); therefore, all tested concentrations were used for comet assay on human lymphocytes.

**Figure 7:**
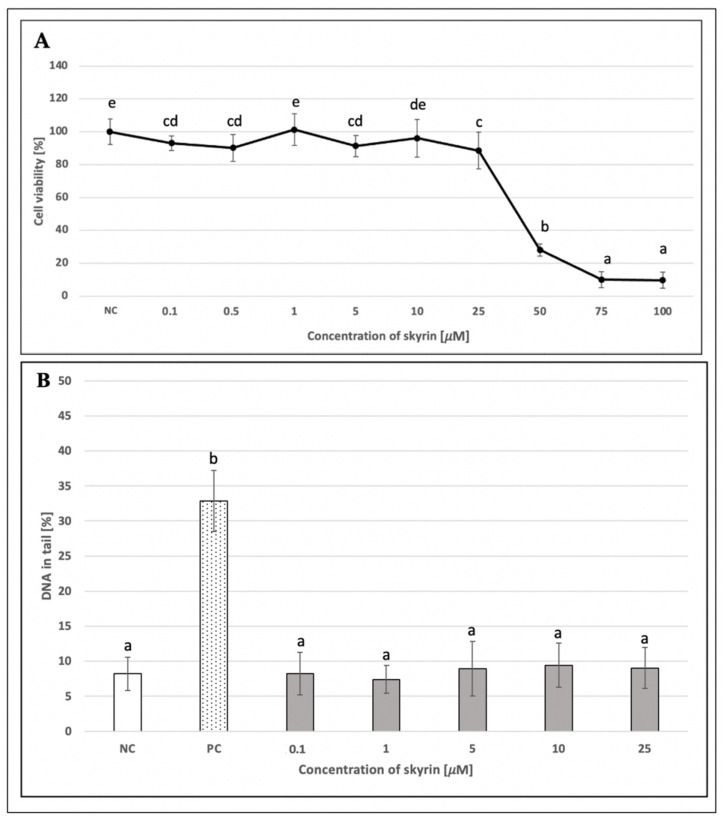
Evaluation of the potential cytotoxic (**A**) and genotoxic (**B**) effects of skyrin (SK) on the HepG2 cell line (24 h treatment) at different concentrations, using the MTT and comet assays. Negative control (NC)—untreated cells; positive control for comet assay (PC)—cells treated with 1 mM hydrogen peroxide. Results were analyzed by ANOVA single-step multiple comparisons of means using LSD tests, and comparisons between the mean values were considered significant at *p* < 0.05. Similar letters represent samples with no statistically significant differences between them. All experimental data in this work are from at least three independent experiments.

**Figure 8:**
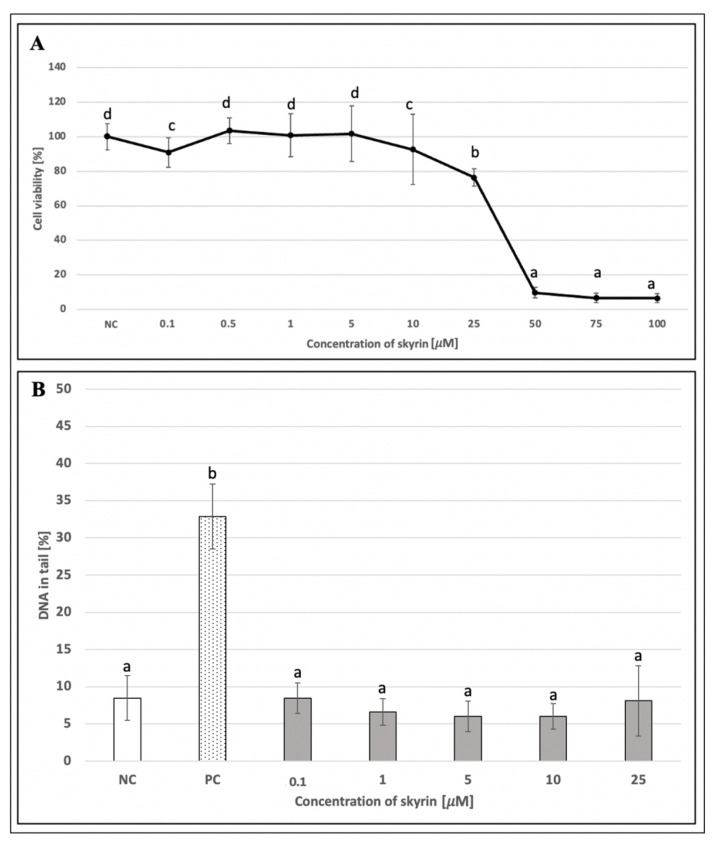
Evaluation of the potential cytotoxic (**A**) and genotoxic (**B**) effects of skyrin (SK) on the HepG2 cell line (24 h treatment followed by washing step and 24 h recovery period) at different concentrations using the MTT and the comet assays. Negative control (NC)—untreated cells; positive control for comet assay (PC)—cells treated with 1 mM hydrogen peroxide. Results were analyzed by ANOVA single-step multiple comparisons of means using LSD tests, and comparisons between the mean values were considered significant at *p* < 0.05. Similar letters represent samples with no statistically significant differences between them. All experimental data in this work are from at least three independent experiments.

**Figure 9:**
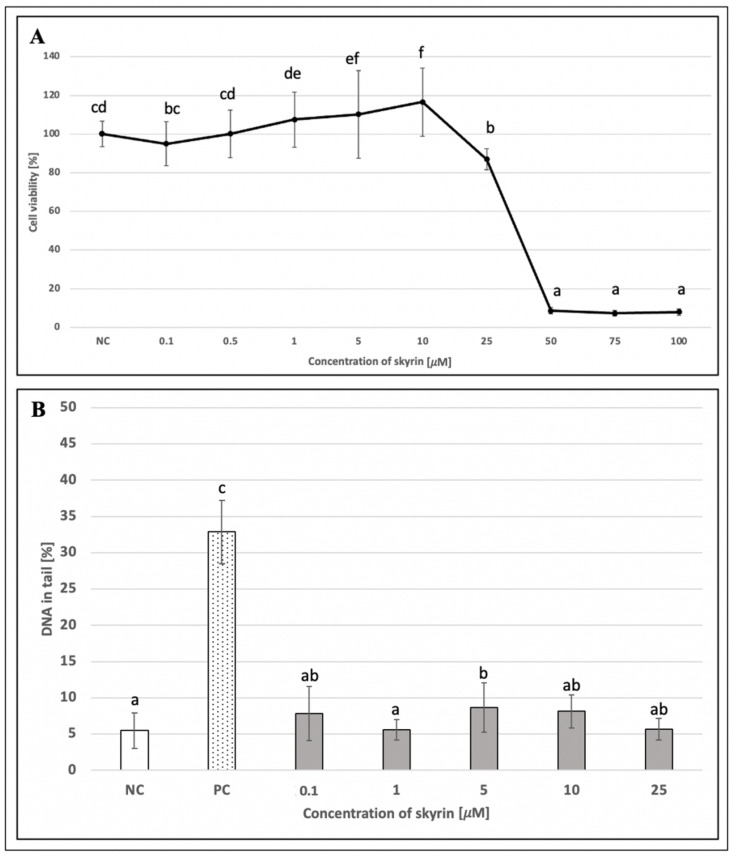
Evaluation of the potential cytotoxic (**A**) and genotoxic (**B**) effects of skyrin (SK) on the HepG2 cell line (48 h treatment) at different concentrations, using MTT and the comet assays. Negative control (NC)—untreated cells; positive control for comet assay (PC)—cells treated with 1 mM hydrogen peroxide. Results were analyzed by ANOVA single-step multiple comparisons of means using LSD tests, and comparisons between the mean values were considered significant at *p* < 0.05. Similar letters represent samples with no statistically significant differences between them. All experimental data in this work are from at least three independent experiments.

MTT assay as a standard colorimetric method for measuring cell viability for culture cell lines was performed using HepG2 cells treated with skyrin in two experimental designs: 24 and 48 h treatments, respectively. In addition, a 24 h treatment, including a washing step and subsequent period for recovery of cells after skyrin treatment (for 24 h), was also implemented. The most significant decrease in viability was observed in samples treated with 50, 75, and 100 μM skyrin in all three treatment schedules (Figure 7A, Figure 8A and Figure 9A). Since the comet assay is not capable of scoring the level of DNA damage in dead cells, these higher concentrations of skyrin (from *Penicillium islandicum*) were not included in the comet assay on HepG2. Other tested concentrations of skyrin did not lead to decreases in viability greater than 20%; therefore, these results are not considered as clinically significant in matters of relation to cytotoxicity.

An alkaline comet assay was performed for the assessment of the potential genotoxic effect of skyrin. The level of DNA damage in human lymphocytes treated with different concentrations of skyrin (0.01–100 μM) remained comparable with the negative control (untreated cells) (Figure 6B). Similar results were obtained in human hepatoma HepG2 cells for all experimental designs. None of the tested skyrin concentrations (0.1–25 μM) showed significant differences in DNA damage compared to the negative control (Figure 7B, Figure 8B and Figure 9B).

### 2.4. Protective Potential of Skyrin against Hydrogen Peroxide

**Figure 10:**
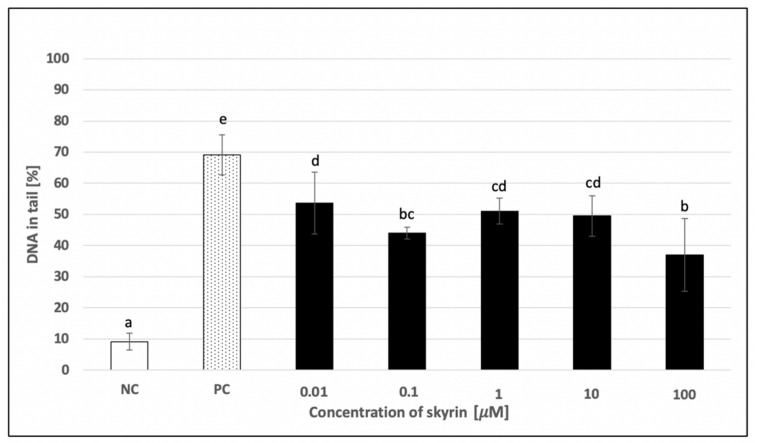
Evaluation of the potential DNA-protective effects of skyrin (SK) on human lymphocytes (1 h treatment) at different concentrations, using the comet assay. Negative control (NC)—untreated cells; positive control for comet assay (PC)—cells treated with 440 μM hydrogen peroxide. Tested samples were treated with a different concentration of skyrin (1 h treatment), and hydrogen peroxide (440 μM) was subsequently added (5 min treatment). Results were analyzed by ANOVA single-step multiple comparisons of means using LSD tests, and comparisons between the mean values were considered significant at *p* < 0.05. Similar letters represent samples with no statistically significant differences between them. All experimental data in this work are from at least three independent experiments.

**Figure 11:**
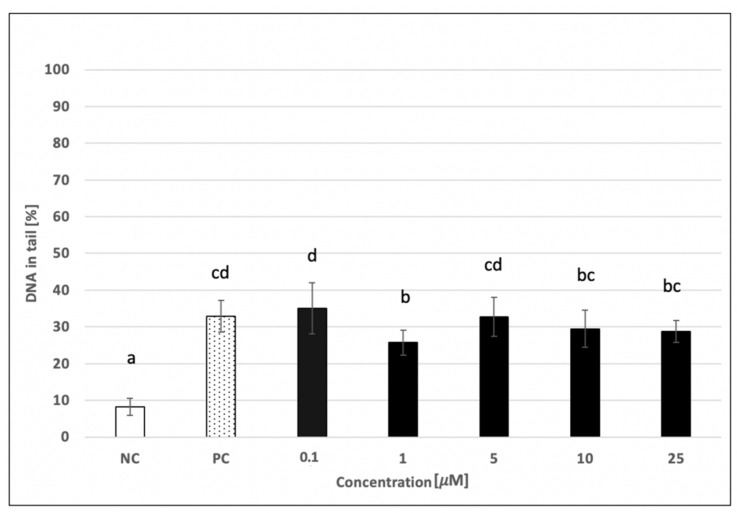
Evaluation of the potential DNA-protective effects of skyrin (SK) on the HepG2 cell line (24 h treatment) at different concentrations using the comet assay. Negative control (NC)—untreated cells; positive control for comet assay (PC)—cells treated with 1 mM hydrogen peroxide. Tested samples were treated with a different concentration of skyrin (24 h treatment), and hydrogen peroxide (1 mM) was subsequently added (5 min treatment). Results were analyzed by ANOVA single-step multiple comparisons of means using LSD tests, and comparisons between the mean values were considered significant at *p* < 0.05. Similar letters represent samples with no statistically significant differences between them. All the experimental data in this work are from at least three independent experiments.

The potential protective activity of skyrin against a damaging agent (hydrogen peroxide) was also investigated using human cells. Significant decreases in hydrogen peroxide-induced DNA damage were observed at all tested concentrations of skyrin using human lymphocytes (Figure 10). The most evident change in DNA damage level was observed in samples treated with 0.1 μM (44 ± 1.95%) and 100 μM (36.93 ± 11.67%) skyrin, compared to samples treated only with 440 μM hydrogen peroxide (69.12 ± 6.39%). Cytotoxic evaluations of the 100 μM skyrin and hydrogen peroxide combination revealed a slight decrease in cell viability (data not shown). Therefore, the change in DNA damage observed in the sample with 100 μM skyrin might be related to higher cytotoxic effects of skyrin in combination with hydrogen peroxide and not to the potential protective effects of skyrin against hydrogen peroxide. DNA protectivity of skyrin was even less noticeable in the HepG2 cancer cell line (Figure 11). Only 1 μM concentration of skyrin significantly decreased DNA damage (25.71 ± 3.39%) compared to the positive control treated with 1 mM hydrogen peroxide (32.87 ± 4.37%). Similar results were obtained for all treatment schedules (24–48 h).

## 3. Discussion

Natural compounds and secondary metabolites represent a great new source of therapeutics due to their wide range of biomedical properties. One of their advantages is a potential ability to act selectively against tumor cells without causing excessive damage to the surrounding tissue [27] or protective capabilities in terms of antioxidant effects [28]. *Hypericum perforatum* L., for which its metabolites display a promising potential in anticancer therapy, is an example of such a therapeutic source. A medically significant substance, hypericin, has shown relevance in photodynamic therapy due to its photoactivation ability [12]. Therefore, the study of the hypericin-related metabolites and its precursors seems to be relevant in order to identify similar effects and verify their safety in biological systems.

Firstly, we focused on the skyrin molecular mode of action in a cell-free system using plasmid DNA and the identification of whether skyrin can induce DNA damage in the form of DNA breaks. The results revealed that skyrin did not cause any form of detectable damage in this model system. DNA-topology assay can be also used for the determination of possible protective effects in the tested compound. In this case, the damaging agent (namely FeSO_4_ × 7H_2_O) was added into samples with plasmid DNA and skyrin. Ferrous ions are known for the production of reactive oxygen species through Fenton reactions in the system and, therefore, the induction of breaks in DNA [29]. Skyrin exhibited DNA-protective effects in a concentration-dependent manner with no damaging effects of ferrous ions observed in the skyrin-containing samples. Similar results were obtained for a structurally similar metabolite, emodin [9]. Since skyrin was able to protect DNA, we further investigated the potential mechanism of its action using a series of spectrophotometric methods. However, skyrin did not show strong antioxidant effects in our experiments since it was not able to either scavenge DPPH^•^ radicals or donate electrons effectively. The chelating activity of skyrin toward transition metal ions was also weak. These results are in contrast with the findings of [30], since they described some antioxidant skyrin activity, namely its ability to scavenge galvinoxyl and hydroxyl radicals, using UV-Vis spectrophotometry and chemiluminescence measurement, respectively. However, in this study a different source of skyrin was used that may have also affected its antioxidant capacity. The radical scavenging activity of mycelial methanol and a water extract solutions prepared from *Ophiocordyceps formosana* was confirmed by [31] using DPPH^•^ radical scavenging assay. The individual components of the extracts were further analyzed by HRMS, with skyrin being identified as one of them. Since these extracts also contained other compounds, the antioxidant potential cannot be attributed solely to skyrin. Similar results to ours were obtained using emodin [9], whereas emodin in analogous concentrations (100 μM and lower) also did not demonstrate antioxidant effects. Since from a chemical perspective skyrin is formed by two molecules of emodin shown to bind/interact with DNA [32,33], we believe that a similar principle might account for the DNA-protective ability of skyrin against the effects of ferrous ions.

Next, the cytotoxic and genotoxic potential of skyrin directly in eukaryotic cells was investigated. The determination of skyrin cytotoxicity was carried out using the trypan blue exclusion test in human lymphocytes and MTT assay in HepG2 cells. Regarding cytotoxicity in non-cancerous cells, skyrin did not show such effects. Since the percentage of lymphocyte viability for all tested concentrations of skyrin remained over 90%, we proceeded to use this concentration range in the comet assay, as well. The cytotoxic effects of skyrin were also investigated using several non-cancerous mammalian cell lines (L929 mouse cell line and BHK(21)C13 hamster cell line). Extracts from *Aschersonia samoensis* containing skyrin showed cytotoxic effects in higher concentrations (ID50 = 284.2 ± 26 mM for L929 cells; ID50 = 92.9 ± 9.5 mM for BHK(21)C13 cells) [34]. However, no significant decrease in metabolite activity after 0.5 and 10 μM skyrin treatment (48 h) was detected using CCD-19Co and CCD-1072Sk colon tissue cell lines [35]. On the other hand, skyrin was able to reduce HepG2 cells viability by approximately 80% at higher concentrations (50–100 μM) in our experiments. These results were observed for all three treatment schedules; therefore, due to their significant cytotoxic effects, the higher concentrations were excluded from the other experiments. The cytotoxicity of skyrin was also detected using a NCI60/ATCC panel of human cancer cell lines, with MIA-PaCa-2 being the most sensitive (IC50 = 50 ± 2.6 μM) after 48 h treatment [36]. Moreover, HL-60 promyelotic cells derived from human leukemia also showed a sensitivity to skyrin ((IC50 = 74 μM) [37]. Using a growth inhibition assay, IC50 values for skyrin treatment (72 h) were estimated when using different cancerous cell lines, namely Calu-1 (IC50 = 26.6 ± 4.6 μM), HeLa (IC50 = 21 ± 6.5 μM), and K562 cells (IC50 = 50.7 ± 9.3 μM) [38]. In addition, a significant decrease in metabolite activity was also detected in HCT116 and HT-29 cancer cell lines after incubation with skyrin. Furthermore, the effects were more prominent after long exposures of cells to skyrin (48 h). Additionally, these concentrations resulted in a 25% decrease in metabolite activity and were 17.6 ± 1.5 μM and 29.4 ± 2.1 μM for HCT116 and HT-29, respectively [35]. The cytotoxic effects of skyrin on HepG2 cells were also described by [34], using concentrations (ID50 = 56.6 ± 25.4 μM) equivalent to ours.

The comet assay is a simple yet effective method for monitoring the occurrence of primary DNA damage in different cell types, including human lymphocytes, isolated from peripheral blood. In this case, no effects of skyrin producing DNA breaks were observed in our study. Interestingly, emodin showed an increase in DNA damage in a concentration-dependent manner before and after irradiation with visible light [9]. The DNA-damaging effects of hypericin, a potential product of skyrin biosynthetic transformation [16], were also analyzed [8]. Before photoactivation, hypericin did not cause increases in primary DNA damage similar to those of skyrin. Concentration-dependent elevations of DNA damage were observed only after the irradiation of hypericin with visible light.

Since skyrin did not show genotoxic potential in non-cancerous cells, we decided to investigate its effects on tumorous HepG2 cells. Skyrin was not able to induce primary DNA damage above 15% in any of the treatment periods (24–48 h). Although the 5 μM concentration of skyrin after the longer exposure period (48 h) was evaluated as statistically significant compared to the negative control, this increase in DNA damage could not be considered clinically significant. DNA fragmentation of cancer cells by skyrin was also investigated using the HL-60 cell line, with electrophoretic analysis of DNA fragments stained by ethidium bromide used to establish the minimal effective dose (37.14 μM) after a 24 h treatment [37]. These results indicate diverse susceptibility of cancer cells to skyrin regarding the induction of DNA damage.

The DNA-protective effects of skyrin using plasmid DNA might also indicate its potential ability to protect DNA in cell systems. For the verification of this hypothesis, a comet assay in the presence of hydrogen peroxide and skyrin was performed. Cells were pretreated with different concentrations of skyrin, with hydrogen peroxide added as a damaging agent after the washing step. In the case of human lymphocytes, skyrin was able to significantly reduce primary DNA damage in these samples. The most prominent decrease in DNA damage was observed with a 100 μM concentration of skyrin. Two possible explanations can be proposed for these results. Firstly, skyrin in higher concentrations may possess DNA-protective effects that were also observed in plasmid DNA by a DNA-topology assay. Another reason for the reduction in DNA damage in skyrin and hydrogen peroxide-treated human lymphocytes might be an elevated cytotoxic effect that potentially overlaps the genotoxicity established by the comet assay. Experiments regarding the possible cytotoxic effects of skyrin with hydrogen peroxide (440 μM) were performed. Cell viability in 100 μM skyrin samples (approximately 78 ± 6.2%) showed mild decreases compared to a negative control (data not shown). However, the value of viable cells did not show a decrease sufficient to cover genotoxicity. Meanwhile, the difference in HepG2 cells treated only with hydrogen peroxide (positive control) and HepG2 cells pre-treated with skyrin and subsequently challenged with hydrogen peroxide was not as prominent as in human lymphocytes.

## 4. Materials and Methods

### 4.1. Tested Compound

Skyrin (SK), chemically C_30_H_18_O_10_, was purchased from Sigma Aldrich (St. Louis, MO, USA). The tested agent was dissolved in pure dimethyl sulfoxide (DMSO, purchased from Serva, Heidelberg, Germany) to prepare a stock solution (1 mg/mL). Tested concentrations were prepared from the stock solutions by diluting them in distilled water (for cell-free methods), phosphate buffer solution (PBS, for methods using lymphocytes), or Dulbecco’s Modified Eagle’s Medium (DMEM, for methods using HepG2 cells).

### 4.2. DNA-Topology Assay

The electrophoretic monitoring of topological changes in the plasmid DNA (pBR322, New England BioLabs, Ipswich, MA, USA) induced by FeSO_4_ × 7H_2_O (Lachema, Brno, Czech republic) was used to detect the DNA-damaging and DNA-protective potential of skyrin, as [39,40] described in detail. In brief, the reaction mixture (final volume of 10 μL) consisted of plasmid DNA (200 ng) and either 1 mM FeSO_4_ × 7H_2_O alone (positive control) or tested concentrations of skyrin (0.0001–100 μM) alone, or combinations of skyrin with 1 mM FeSO_4_ × 7H_2_O. 0.1 M phosphate buffer (1 M KH_2_PO_4_, 1 M K_2_HPO_4_, both purchased from Sigma Aldrich, St. Louis, MO, USA; pH 7.4) was added to all samples and they were incubated 50 min in the dark at room temperature. An analysis of changes in the DNA topology caused by DNA breaks was carried out by gel electrophoresis (in 1% agarose for 90 min/100 V). The DNA was stained with GelRed dye (1 mg/mL, Sigma Aldrich, St. Louis, MO, USA) and visualized by UV illumination (UV Transilluminator MiniBISPro, DNR Bio Imaging Systems Ltd., Neve Yamin, Israel). Increases in DNA strand breakage were assayed by measuring the conversion of supercoiled DNA, form III, to relaxed circular (I) and linear forms (II). Densitometric quantification of plasmid topology forms (%) was carried out in the ImageJ 1.53c program (Wayne Rasband, National Institute of Health, Kensington, MD, USA).

### 4.3. Reducing Power Assay

The reducing capacities of the skyrin were determined according to [41]. In this assay, the yellow color of the test solution is changed to various shades of green and blue, depending on the reducing power of the studied compound. The presence of reducing agents (antioxidants) induces the conversion of the Fe^3+^/ferricyanide complex into ferrous forms. In brief, different concentrations (0.0001–100 μM) of skyrin and gallic acid (used as a positive control, Sigma Aldrich, St. Louis, MO, USA) (200 μL) were mixed with 500 μL of a phosphate buffer (0.2 M, pH 6.6) and 500 μL of potassium ferricyanide K_3_[Fe(CN)_6_] (1%, Lachema, Czech republic). The mixtures were incubated at 50 °C for 20 min. Trichloroacetic acid (500 μL, 10%, Avondale Laboratories, Oxfordshire, UK) was added to each sample and centrifuged at 600× *g* (MiniSpin^®^ Centrifuge Eppendorf, Hamburg, Germany) for 10 min. Finally, the upper layer of supernatant (500 μL) was mixed with 500 μL of distilled water and 100 μL of FeCl_3_ (0.1%, Reanal, Budapest, Hungary), and the absorbances were measured at 700 nm (GENESYS 10 Bio, Spectronic, Thermo Fisher Scientific, Waltham, MA, USA). The higher absorbance of the reaction mixture indicates the higher reducing power of the tested compound.

### 4.4. DPPH^•^ Radical Scavenging Activity

Different concentrations of skyrin were investigated for potential radical scavenging activity using a modified DPPH^•^ assay [42]. DPPH^•^ (1,1-diphenyl-2-picrylhydrazyl) is a stable free radical. At the radical state, the methanolic solution of this compound is dark purple (absorbs light at a wavelength of 517 nm). When DPPH^•^ reacts with an antioxidant, by providing hydrogen atoms or by electron donation, it is reduced to the molecular form with a yellow color. In brief, a methanol solution of a DPPH^•^ radical (Sigma Aldrich, St. Louis, MO, USA) with a concentration of 0.1 mM (950 μL) was added to 50 μL of various concentrations of skyrin or gallic acid (Sigma Aldrich, St. Louis, MO, USA), used as a positive control. The experiments were carried out at room temperature. The samples were incubated 30 min in the dark, and a decrease in absorbance at 517 nm was afterward measured using a spectrophotometer (GENESYS 10 Bio, Spectronic, Thermo Fisher Scientific, Waltham, MA, USA). The DPPH^•^ scavenging activities of skyrin and gallic acid were then expressed as percentages of DPPH^•^ scavenging activity using the following formula:

scavenging of DPPH^•^ radicals (%) = [(A_control_ − A_sample_)/A_control_] × 100

where A_control_ is the absorbance of the control reaction, containing all reagents except the tested compounds, and A_sample_ is the absorbance of tested compounds. Pure methanol (CentralChem, Bratislava, Slovakia) was used as a blank.

### 4.5. Fe^2+^-Chelating Activity Assay

The chelating activity of skyrin, as one of the possible mechanisms of DNA-protectivity, was estimated. The potential antioxidants are able chelate the transition metals and, in this manner, prevent the decomposition of the hydroperoxide and Fenton-type reactions. If the tested compound possesses antioxidant activity, it may chelate the ferrous ions from the ferrous chloride in the samples. The remaining ferrous ions form ferrous–ferrozine complexes. The intensity of this ferrous–ferrozine complex formation depends on the chelating capacity of the compound and results in a reduction in color. The chelating activity of skyrin toward ferrous ions was conducted as described previously [43]. Briefly, 50 μL of a 2 mM FeCl_2_ solution (Slavus, Bratislava, Slovakia) was added to aliquots of 100 μL of skyrin solution and 900 μL of distilled water. The reaction was initiated by adding 200 μL of 5 mM ferrozine solution (Sigma Aldrich, St. Louis, MO, USA) to the samples. After incubation (10 min) in the dark at room temperature an absorbance at 562 nm was measured (GENESYS 10 Bio, Spectronic, Thermo Fisher Scientific, Waltham, MA, USA). A reaction mixture containing 100 μL of solvent (distilled water) instead of the sample solution served as a negative control. The chelating activity of the samples was calculated according to the following formula:

inhibition of ferrous-ferrozine complex formation (%) = [(A_control_ − A_sample_)/A_control_] × 100

where A_control_ is the absorbance of the control reaction, containing all reagents except the tested compounds, and A_sample_ is the absorbance of the tested compounds. Distilled water was used as a blank.

### 4.6. Trypan Blue Exclusion Test

The dye exclusion test was used to determine the number of viable human lymphocytes present in a sample after treatment and, thus, to identify the potential cytotoxic effect of the tested metabolite. This method is based on the fact that live cells possess intact cell membranes that exclude certain dyes, such as trypan blue, whereas dead cells do not and, therefore, remain stained. The cell viability evaluation was performed according to [44] with several modifications. The human lymphocytes were isolated using the finger prick method. Cells were separated from the blood sample using the Histopaque gradient medium (Sigma Aldrich, St. Louis, MO, USA) and a buffy coat was used for the next procedure. After isolation, the cells were treated with skyrin (total volume of sample was 1 mL) for 1 h at 25 °C. Negative control samples remained untreated and left in fresh PBS (137 mM NaCl (CentralChem, Bratislava, Slovakia), 2.7 mM KCl (Sigma Aldrich, St. Louis, MO, USA), 8 mM Na_2_HPO_4_, 2 mM KH_2_PO_4_ (both purchased from CentralChem, Bratislava, Slovakia); pH 7.4) for 1 h at 25 °C. After the treatment phase, a washing step using PBS was performed, and the cell suspension was mixed with trypan blue dye (Slavus, Bratislava, Slovakia) (0.4% solution in PBS, pH 7.2 to 7.3) in a 4:1 ratio. Cells were incubated with the dye for 2 min and counted with a hemocytometer at a magnification of 100×. The percentage of viable cells was calculated according to the following formula.

percentage of viable cells = (number of viable cells/number of total cells) × 100

### 4.7. Cell Culture

The HepG2 malignant cell line (human hepatocellular carcinoma cells) was a generous gift from Prof. Peter Eckl (University of Salzburg, Salzburg, Austria). The cells were cultured in DMEM medium supplemented with 10% fetal bovine serum and antibiotics (penicillin 200 U/mL, streptomycin 100 μg/mL). The cells were cultured on plastic Petri dishes and plates at 37 °C in a humidified atmosphere of 5% CO_2_. All chemicals were purchased from Gibco Invitrogen (Waltham, MA, USA).

### 4.8. MTT Assay

HepG2 cells were seeded into the series of 96-well plates at a density of 2 × 10^4^/well, and cultured in a DMEM medium. The exponentially growing cells were then pre-incubated in the presence of skyrin (0.1–100 μM) or without compound (control) for 24–48 h and used for testing the cytotoxicity by the MTT assay according to [45]. The MTT test is a colorimetric method for measuring the activity of mitochondrial reductases that transform yellow MTT (3-(4,5-dimethylthiazol-2-yl)-2,5-diphentltetrazolium bromide) to purple insoluble formazan. This reduction occurs only when reductase enzymes are active; therefore, the level of conversion is often used as a measure of viable cells. In our experiments, we incubated the properly treated HepG2 cells in 50 μL of MTT solution (1 mg/mL, Sigma Aldrich, St. Louis, MO, USA) and 100 μL of DMEM medium (Gibco Invitrogen, Waltham, MA, USA) for 4 h at 37 °C. For each sample, at least six wells were used. Then, the MTT solution was removed, 100 μL of DMSO (Serva, Heidelberg, Germany) was added to each well, and the plates were placed on an orbital shaker for 15–20 min to completely dissolve the formazan crystals. Absorbance at 540 nm was measured using an xMarkTM microplate spectrophotometer (Bio-Rad Laboratories, Inc., Hercules, CA, USA) and background absorbance at 690 nm was subtracted. The viability of the HepG2 cells was calculated using the following formula.

percentage of viable cells = (A_treated oells_/A_control cells_) × 100

### 4.9. Comet Assay Using Human Lymphocytes

The comet assay was performed according to [46] with modifications described by [10]. The human lymphocytes were isolated from the peripheral blood using the Histopaque gradient medium (Sigma Aldrich, St. Louis, MO, USA), and the cells were resuspended in 1% low melting point agarose (Roth, Karlsruhe, Germany) in PBS (137 mM NaCl (CentralChem, Bratislava, Slovakia), 2.7 mM KCl Sigma Aldrich (St. Louis, MO, USA), 8 mM Na_2_HPO_4_, 2 mM KH_2_PO_4_ (both purchased from CentralChem, Bratislava, Slovakia); pH 7.4). A volume of 120 μL of the cell suspension was layered onto 1% normal melting point agarose (Roth, Karlsruhe, Germany) pre-coated slides. Subsequently, cover slips were placed on the slides to ensure even spreading. After gel solidification, the cells were treated with different concentrations of skyrin (0.01–100 μM) for 1 h at 25 °C. Samples treated with 440 µM hydrogen peroxide (Sigma Aldrich, St. Louis, MO, USA) were used as a positive control (5 min). For negative control, the cells were left untreated in fresh PBS for 1 h at 25 °C. For the investigation of DNA-protective effects, samples were pre-treated with skyrin (1 h), and after the washing step, they were challenged with 440 µM hydrogen peroxide (5 min). The cells were lysed by immersing the slides in a lysis solution (2.5 M NaCl (CentralChem, Bratislava, Slovakia), 100 mM Na_2_EDTA, 10 mM Tris-HCl and 1% Triton X-100 (all three from Sigma Aldrich, St. Louis, MO, USA); pH 10) at 4 °C for 1 h. After lysis, the slides were transferred into an electrophoretic chamber containing a fresh alkaline electrophoretic solution (300 mM NaOH, 1 mM Na_2_EDTA, both purchased from Sigma Aldrich, St. Louis, MO, USA; pH > 13) and left for 15 min at 4 °C to allow the DNA to unwind. The electrophoresis was launched for 30 min at 4 °C at 25 V and 260–320 mA. The slides were neutralized by washing in PBS (5 min) and dH_2_O (5 min). Subsequently, the samples were stained with ethidium bromide dye (20 μg/mL, Sigma Aldrich, St. Louis, MO, USA). Using a fluorescence microscope (OLYMPUS BX 51) and green excitation filter UMWIG3, 100 random nucleoids in each sample were scored at magnification 400×. Each comet was scored from 0 to 4 (0 = undamaged, 4 = >80% DNA in the tail) depending on the relative intensity of DNA fluorescence in the comet tail, and the final percentage of DNA damage was calculated from the total score for each sample.

### 4.10. Comet Assay Using HepG2 Cell Line

For the assessment of DNA-damaging by skyrin, a concentration range of 0.1−25 μM was selected for the 24–48 h treatment of HepG2 cells (their viability was above 70%). In the case of the DNA-protective effects of skyrin, 5 min post-treatment with H_2_O_2_ was applied. Comet assay was performed according to [47,48] with minor adjustments. In brief, microscopic slides were coated with 1% normal melting point agarose (Roth, Karlsruhe, Germany) at least 24 h prior to the experiments. The tested HepG2 cells (untreated or treated with skyrin) at a density of 3 × 10^4^ cells/50 μL of 0.75% low melting point agarose (Gibco Invitrogen, Waltham, MA, USA) in PBS were placed on pre-coated microscopic slides and covered with a cover slip. After the solidification of the gels (15 min), the cover slips were removed and the slides were placed in a lysis mixture (2.5 M NaCl (CentralChem, Bratislava, Slovakia), 100 mM Na_2_EDTA, 10 mM Tris, 1% Triton X-100 (all three from Sigma Aldrich, St. Louis, MO, USA); pH 10) for 1 h at 4 °C. Samples were consequently transferred to an electrophoresis solution (300 mM NaOH, 1 mM Na_2_EDTA, both purchased from Sigma Aldrich (St. Louis, MO, USA); pH > 13) for unwinding (40 min) at 4 °C and were then subjected to electrophoresis at 25 V (current adjusted to 0.3 A) for 30 min at 4 °C. Finally, the slides were twice neutralized with 400 mM Tris-HCl (pH 7.5, Serva, Heidelberg, Germany) for 10 min and stained with ethidium bromide (5 μg/mL, Sigma Aldrich, St. Louis, MO, USA). For the evaluation of DNA damage as a percentage of DNA in the tail of the comets, at least 100 ethidium bromide-stained nucleoids were scored for each slide with a Zeiss fluorescent microscope and automated computerized image analysis Metafer 3.6 system (MetaSystems GmbH, Altlussheim, Germany).

### 4.11. Statistical Analysis

The data obtained were analyzed using Statgraphics Centurion XV v. 15.2.05 (StatPoint, Inc., Warrenton, VA, USA) and Excel (Microsoft Office 2007). The treatment effects of skyrin were analyzed by ANOVA single-step multiple comparisons of means using LSD tests, and comparisons between the mean values were considered significant at *p* < 0.05. All experimental data in this work are from at least three independent experiments.

## 5. Conclusions

Our findings indicate that skyrin may possibly protect the DNA of non-cancerous cells more effectively than the DNA of tumor cells. Such an effect of skyrin may be potentially applicable in cancer therapy. To the best of our knowledge, this is the first study aimed at the potential protective effects of skyrin in a cellular system. Therefore, further research regarding the mechanism of skyrin action in cells is needed.

## Data Availability

Not applicable.
